# T cell-specific inactivation of mouse CD2 by CRISPR/Cas9

**DOI:** 10.1038/srep21377

**Published:** 2016-02-23

**Authors:** Jane Beil-Wagner, Georg Dössinger, Kilian Schober, Johannes vom Berg, Achim Tresch, Martina Grandl, Pushpalatha Palle, Florian Mair, Markus Gerhard, Burkhard Becher, Dirk H. Busch, Thorsten Buch

**Affiliations:** 1 Institute for Medical Microbiology, Immunology and Hygiene, Technische Universität München, Germany; 2 Institute of Laboratory Animal Science, University of Zurich, Schlieren, Switzerland; 3 Max-Planck-Institute for Plant Breeding Research, Cologne, Germany; 4 Department of Biology, Albertus-Magnus University, Cologne, Germany; 5 Institute of Experimental Immunology, University of Zurich, Zurich, Switzerland

## Abstract

The CRISPR/Cas9 system can be used to mutate target sequences by introduction of double-strand breaks followed by imprecise repair. To test its use for conditional gene editing we generated mice transgenic for CD4 promoter-driven Cas9 combined with guide RNA targeting CD2. We found that within CD4^+^ and CD8^+^ lymphocytes from lymph nodes and spleen 1% and 0.6% were not expressing CD2, respectively. T cells lacking CD2 carryied mutations, which confirmed that Cas9 driven by cell-type specific promoters can edit genes in the mouse and may thus allow targeted studies of gene function *in vivo*.

Genome modification in the mouse has become an essential research tool for addressing gene function *in vivo*. Since 1987, targeted mutagenesis in the mouse has been performed by manipulating embryonic stem (ES) cells *in vitro*^1^. Chimeric mice that were partially derived from these ES cells were used to find new, manipulated mouse lines. As an extension to this technology, recombinases such as Cre are used to avoid the use of resistance genes, to allow embryonic-lethal mutations and to make inducible, cell-type specific knockouts and knock-ins^2^,^3^,^4^. While, recombinase-mediated conditional gene targeting has drastically widened the scope of the technology, it still relies on ES cells for introduction of the recombinase targets such as loxP sites. Further, mouse lines carrying a loxP-containing allele need to be intercrossed with a Cre transgene to address target gene function. Altogether, the minimal time for generation of such conditional mutants is more than a year. Recently, a paradigm shift in germline mutagenesis has taken place through DNA editing by designer nucleases. Zinc finger nucleases, Tal effector nucleases, and the CRISPR/Cas9 system have facilitated the generation of gene-modified animals without the use of ES cells^5^,^6^,^7^,^8^,^9^. These designer nucleases can be tailored to introduce single- or double-strand breaks into target genes, which are repaired through cell-intrinsic repair mechanisms. Non-homologous end joining-mediated repair leads to insertions or deletions^10^ and homologous recombination in the presence of a donor fragment facilitates introduction of defined mutations^11^. In the mouse, designer nuclease-mediated mutagenesis is achieved through injection of nuclease-encoding plasmids, RNA or protein into zygotes followed by screening of the founder animals^5^,^6^. Recently, the Clustered Regularly Interspaced Short Palindromic Repeats (CRISPR) in combination with CRISPR associated gene 9 (Cas9) system has surpassed the other designer nucleases in terms of efficiency and flexibility. It has proven to allow both insertions/deletions and specific targeted mutagenesis in the germline of the mouse^8^,^12^,^13^,^14^ and in human cells^14^,^15^. The original CRISPR/Cas9 system from *Streptococcus pyogenes*^16^ was modified for application in molecular biology and now relies on a single 102 bp long guide RNA (gRNA), of which 20 bp determine the target sequence^17^. Since, the exact choice of gRNA is subject to further restrictions, such as presence of the *Protospacer Adjacent Motif* (PAM) sequence (NGG) and uniqueness within the target genome, online tools have been developed for designing functionally competent gRNAs^18^,^19^. The CRSIPR/Cas9 system has been used to modify genes in the germline by transient expression in the zygote and was found to facilitate somatic mutations in the mouse when expression of the system was under control of the Cre/loxP system and gRNA was delivered through viruses or transfection^20^. Only recently, it was shown in mice that doxycycline-induced Cas9 allows conditional *in vivo* gene editing^21^. In *Drosophila melanogaster*, Cas9 was shown to enable efficient cell-specific gene editing after its placement under a tissue-specific promoter^22^. However, direct gRNA/Cas9-mediated conditional gene editing using cell-type specific promoters similar to conditional mutagenesis by Cre has been so far not reported for the mouse (Fig. 1a). Such an approach would allow single-step analysis of gene function within a cell lineage circumventing tedious gene targeting and crossing necessary for Cre/loxP-mediated conditional gene ablation.

To test whether gRNA/Cas9 could drive cell-type specific mutagenesis in the mouse, we placed Cas9 under control of a CD4 promoter (CD4dsCas9), thus initiating the expression in T lymphocytes during their maturation in the thymus (Fig. 1b)^23^. In a second construct, we placed a gRNA expression cassette under control of a U6 promoter (U6gRNA(CD2)). The gRNA was directed against a target sequence in exon 2 of CD2 (Fig. 1b), an easily detectable surface marker found on all T cells. In addition, we chose this target because its deficiency does not influence cell viability^24^ and does not lead to changes in the cell’s phenotype, that could impair this proof of concept study. The two constructs were co-injected into the pronuclei of oocytes of FVB/N mice and offspring were screened for presence of the transgenes. Two transgenic founders carrying both constructs were identified (Fig. 1c). We also obtained two founders carrying solely the Cas9 and two carrying solely the gRNA construct (data not shown). Further analysis of peripheral blood by flow cytometry revealed that in one of the double-transgenic founders a fraction of CD4^+^ (0.4%) and CD8^+^ (0.5%) cells lacked expression of CD2 (Fig. 2). No such populations were found in wildtype controls (data not shown), the other double-transgenic (Suppl. Fig. 1) or Cas9 and gRNA single-transgenic founders (Fig. 2). Furthermore, we analyzed pooled lymph nodes and spleen cells of two double transgenic F1 mice as well as of two wildtype littermates to verify the initial blood analysis. Here we observed that 1% of CD4^+^ and 0.6% of CD8^+^ T cells were not expressing CD2 anymore (Fig. 3). A transgene copy number analysis by real-time PCR was performed for this transgenic line and indicated numbers of integrated Cas9 being 26 and 41 and of the gRNA of 12 and 6/7 (Fig. 4a). In addition, CD4-Cas9 mRNA expression was found solely in T cells and thymocytes but not B cells of double transgenic (dTG) mice or wildtype thymocytes (Fig. 4b). U6-driven gRNA(CD2.0) expression was detected in total spleen and thymus of dTGs but not in WT controls (Fig. 4c). To confirm that the CD2 locus was edited by gRNA/Cas9, we single cell-sorted T cells lacking CD2 and sequenced a fragment around the target site after single cell PCR amplification and cloning (Fig. 5a). We observed that eight out of the nine sequenced CD2^−^CD4^+^ T cells carried a mutation in the amplicon (Fig. 5b). In CD2^−^CD8^+^ T cells two of the three amplicons were wildtype and one mutated (Fig. 5b). In all amplicons, we only found a single sequence, either mutated or wildtype. This may be either result of a technical bias leading to amplification of only one allele or alternatively an outcome of repair of the second allele by homologous recombination using an already mutated donor allele. In all 17 amplicons from CD2^+^ T cells as well as nine amplicons from CD19^+^ B cells, we obtained solely unmutated sequences (Fig. 5b). As an additional control, we performed single cell PCR on FVB/N wildtype CD4^+^ and CD8^+^ T cells and sequenced the PCR products, again yielding solely wildtype sequences (Fig. 5b). Sequence analysis of the mutations found within the amplicons from CD2^−^ T cells revealed deletions spanning up to 19 bp and insertions of one to four base pairs, five were found as out of frame and one in frame (Fig. 5c). Two mutations were repeatedly found (Fig. 5c).

Even though we observed CD2^−^ T cells in a gRNA/Cas9 double-transgenic situation, we were surprised that the number of these cells was extremely low. This may have been a specific result of this particular founder due to expression variegation or may constitute a general problem of the approach. We therefore analyzed the T cell compartments in our different transgenic mice in greater detail. The ratio of CD4^+^ TCRβ^+^ and CD8^+^ TCRβ^+^ T to CD19^+^ B cells was found to be slightly increased in double-transgenic (8 (CD4^+^ TCRβ^+^) and 2.2 (CD8^+^ TCRβ^+^)) versus Cas9 single (4.8 (CD4^+^ TCRβ^+^) and 1.4 (CD8^+^ TCRβ^+^)) and gRNA transgenic animals (4.9 (CD4^+^ TCRβ^+^) and 1.8 (CD8^+^ TCRβ^+^)), thus ruling out a general counter-selection of CD4^+^ and CD8^+^ T cells. Since expression of nucleases such as Cre recombinase were reported to lead to apoptosis in situations of high proliferative activity^25^ and also for Cas9 toxic *in vivo* effects were described^22^, we analyzed CD4^+^ and CD8^+^ T cells for cell death (Suppl. Fig. 2a,b). We obtained no evidence of an increased number of dead cells in lymphocytes of the gRNA/Cas9 double-transgenic situation compared to Cas9 or gRNA single transgenic mice as well as wildtype mice. Thus, in our model we cannot find evidence for genotoxicity of gRNA and Cas9. Another important point regarding the use of CRISPR/Cas9 for *in vivo* and *ex vivo* use are off-target effects. Whereas some groups report high specificity of CRISPR/Cas9^26^,^27^ others show high frequency of off-target effects^28^,^29^. Several groups found that off-target effects can be reduced by the use of optimized gRNAs^28^,^30^,^31^. In our case off-target effects were not analyzed because our on-target frequency of modification was already low^32^.

Taken together, our data indicate that gRNA/Cas9 can be used to mutate target genes in specific cell lineages in the mouse. The frequency of such mutations in our system was low. We excluded that this was result of the actual gene deficiency by using a target whose absence was shown to have no effect over T cell survival^24^. Also, we did not find evidence that nuclease activity would increase cell death and thereby remove the edited cells from the population, which could explain the low frequency of mutations. One reason for inefficient abrogation of CD2 expression may, of course, be a feature of this particular transgenic founder. Defined and characterized Cas9-driver lines that are crossed to gRNA lines may overcome such problems in the future. To address this point we analyzed gRNA expression and Cas9 expression on mRNA and protein level. gRNA was expressed in both spleen and thymus. However, while we clearly detected Cas9 mRNA, we failed to see expression of the protein (0.25 ng Cas9 protein detection limit, data not shown). Thus, low protein expression levels in our particular founder line may contribute to the low efficiency of the system. To further address whether or not the efficiency of the used gRNA contributed to the observed low mutation frequencies we compared by *in vitro* digestion of a PCR product the used gRNA (gRNA(CD2.0) to three other gRNAs (gRNA(CD2.1,CD2.2,CD2.3)) targeting the same region (Fig. 6). We found that gRNA(CD2.1) showed almost no cutting and gRNA(CD2.2) complete digest. The gRNA(CD2.0) used for generating the transgenic mice presented with good but not complete cutting efficiency, along with gRNA(CD2.3) (Fig. 6). It thus appears unlikely that the low *in vivo* efficiency of CD4-Cas9/gRNA(CD2.0) is sole result of suboptimal gRNA performance. Methylation of target loci as factor contributing to efficiency of Cas9-mediated genome editing has been also discussed and assessed, though with contradictory results^18^,^32^, but chromatin structure in general appears to influence Cas9 binding^32^,^33^. Interestingly, Cre-induced Cas9 expression from within the *gt(ROSA)26Sor* locus combined with viral gRNA expression also resulted in only infrequent gene editing in 0.1% to 1.8% of lung cells^20^ while a recent report of doxycycline-regulated Cas9-mediated gene editing showed significantly higher frequencies of mutated cells^21^. gRNA library transduction experiments in EL4 cells, a mouse thymic cell line, was found to also lead to only 0.94–3.60% mutant cells when analyzing different surface markers^34^. Furthermore, we were not able to efficiently mutate CD2 in T cell transfection experiments (data not shown). While certainly the evidence is still anecdotal, T cells may be difficult to modify by CRISPR/Cas9. One way to increase the frequency of gene editing events leading to inactivation of gene functionality may be the use of multiple gRNAs towards the same target. Yet, even in such an approach only 2/3 of alleles will carry an out-of-frame mutation. CRISPR/Cas9-mediated *in vivo* targeted mutagenesis may thus work best when a critical region, such as an enzymatic site, is functionally compromised even through in-frame in/del mutations. Nevertheless, also in the here-described simple version conditional DNA editing is extremely useful. It allows rapid assessment of cell-type specific phenotypic changes in a time frame incomparable to classical conditional mutagenesis, especially when mutant cells can be easily identified for phenotypic analysis. Additionally, for the investigation of onco- and tumor suppressor genes the observed low editing frequency may prove to be advantageous. Taken together, this proof of concept study shows that direct conditional gene editing by gRNA/Cas9 transgenesis may extend the toolbox for genetic studies in the mouse.

## Methods

Methods were carried out in accordance with the approved guidelines.

### Animal Models

hCas9 (a gift from George Church (Addgene plasmid # 41815))^15^ was PCR-amplified and placed into the second exon of CD4 in a construct (CD4-DEPE, gift from Marc Schmidt-Supprian) consisting of the CD4 promoter, the distal and the proximal enhancer, exon 1 and parts of exon 2 but lacking the intronic silencer, thus supporting expression in all αβ T cells. Subsequently, the plasmid was digested with NotI. The Crispr design tool at crispr.mit.edu was used to identify the protospacer specific for CD2 Exon 2 (5′-GACTAGGCTGGAGAAGGACC-3′), which was cloned via BbsI into a modified px330 vector (px330ccdBChR), thus replacing a ccdB/chloramphenicol cassette. The transgene was cut out by BciVI and XbaI. Both constructs were injected into FVB/N oocytes. The founders were screened by PCR using the following primers: CD4 Cas9 typ fwd: 5′- tgc tca caa ccc ttt agt tt-3′, CD4 Cas9 typ rev: 5′-ctt ttt atc ctc ctc cac c-3′ (product length: 835bp); U6 fwd: 5′-gag ggc cta ttt ccc atg att cc-3′, T7 gRNA rev: 5′-gca cgc gct aaa aac gga-3′ (product length: 407bp). Animals were kept in barrier-SPF level animal facilities at Technische Universität München according to German Animal Protection Law. Experiments were conducted under the license number 55.2-1-54-2532-2-12 and approved by the Regierung von Oberbayern.

### Transgene copy number determination

We estimated genomic transgene abundance by performing a RT-PCR using SsoFast EvaGreen (BioRad) with following primers: Cas9F2: 5′-aag aga acc cga tca acg a-3′, Cas9R2: 5′-aga tta cca aac agg ccg t-3′; Cas9F3: 5′-gcg cta ggc tgt cca aat-3′, Cas9R3: 5′-att taa agt tgg ggg tca gcc-3′; gRNA(CD2.0)F2: 5′-tgg aaa gga cga aac acc ga-3′, gRNA(CD2.0)R2: 5′-cac gcg cta aaa acg gac-3′; CD8aFb: 5′-caa gga agc aag tgg tat gaa-3′, CD8aRb: 5′-ttt cca gat tta ccg tac cg-3′; TCRaF: 5′-tcc ggc caa acc atc tgt-3′ and TCRaR: 5′-cgc tgg ggg aga tga cta t-3′. A linear regression was performed, in which the dependent variable, the (decadic) logarithm of the amount of DNA used in each PCR experiment was explained by the mean Ct-value (number of PCR cycles until detection level) in replicate experiments and gene-specific primer pairs were used.


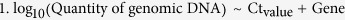


**Figure 1:**
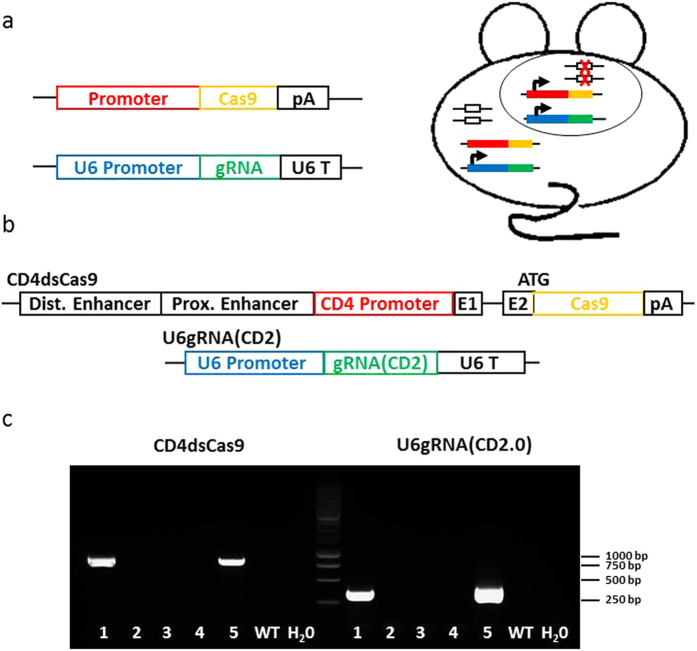
Conditional gene editing. (**a**) Scheme of the concept of conditional gene editing. In the Cas9 driver strain, the nuclease is placed under control of a cell type or lineage specific promoter. The gRNA construct is driven by the ubiquitous U6 promoter. Both transgenes are co-injected into oocytes. In double-transgenic animals, cell-type specific gene deletions are induced. (**b**) Scheme of constructs used for the CD4dsCas9/U6gRNA(CD2) mouse strain. The two used linearized plasmids are shown. First, distal and proximal enhancer, CD4 promoter followed by exon 1, part of exon 2 and Cas9 with a PolyA at the end. Second, the U6 promoter driven gRNA specific for CD2 followed by the U6 terminator (U6 T). (**c**) PCR analysis of tail biopsies for presence of CD4dsCas9 (835 bp amplicon) (lanes 1 and 5) and U6gRNA(CD2.0) (407 bp amplicon) (lanes 1 and 5) by PCR. DNA from a wildtype (WT) mouse as well as H_2_0 were run as a negative control. 2, 3 and 4 were non-transgenic litter mates.

Here, “Ct value” is a continuous variable, and “Gene” is a nominal variable with 5 distinct values coding for the 5 primer pairs used (“CD8a”,“TCRa”,“gRNA CD2.0”,“Cas9 2”,“Cas9 3”). As a result, we obtain one coefficient for “Ct_value_”, β_Cvalue_, and 5 coefficients for “Gene”, β_CD8a_, β_TCRa_, β_gRNA(CD2.0)_ , β_Cas9_2_, β_Cas9_3_ (one of the latter 5 is redundant and set to zero. For our purpose, it is irrelevant which one is chosen). As a result, we obtain a regression line for each primer pair (see plots Fig. 1d). The difference in the coefficients for each factor level of “Gene” is the log10 of the fold difference in genomic abundance of the corresponding amplicons (genes). We chose, the mean 2. γ = (β_CD8a_ + β_TCRa_)/2 of the coefficients β_CD8a_ and β_TCRa_ as reference value, corresponding to two genomic copies of an amplicon. The genomic abundance of gRNA CD2.0 was then estimated as 3. 2*10^(γ-β_TCRa_) (the factor 2 accounts for the two copies of the reference genes), and the genomic abundance of Cas9 was estimated as 4. 2*10^(γ – (β_Cas9_2_ + β_Cas9_3_)/2). In the latter case, we averaged the two abundance measurements for Cas9. Calculations were carried out in R (Version 3.2.0)^35^.

### RT-PCR

The determination of CD4-Cas9 expression was performed on MACS-isolated B and T cells using mouse CD19 microbeads and mouse Pan T Cell Isolation Kit II according to manufacturer’s instruction (Miltenyi Biotec). RNA was isolated using RNeasy Mini Kit (Qiagen) and transcribed with iScript cDNA Synthesis Kit (BioRad). RT-PCR was performed with SsoFast EvaGreen Supermix (BioRad) and template-free negative controls were included. For the quantification of U6-gRNA(CD2.0) the total RNA of thymus and spleen was isolated using RNeasy Mini Kit (Qiagen) and reverse-transcribed with Moloney Murine Leukemia Virus Reverse Transcriptase RNase H− Point Mutant (Promega) according to the manufacturer’s instructions. RT-PCR was performed with KAPA SYBR FAST qPCR Universal Master Mix (Peqlab) and template-free and reverse transcriptase-free negative controls were included. Both transcript abundances were assessed using the Bio-Rad CFX384 systems and following primers were used: CD4-Cas9 F: 5′-agc cct cat ata cac aca cct-3′, CD4-Cas9 R: 5′-acg ctg ttt gtg ccg ata-3′; CD4 F: 5′- aga act ggt tcg gca tga ca-3′, CD4 R: 5′-aag gag aac tcc gct gac-3′; CD2 F: 5′-ccc atg att cct tca tat ttg ca-3′ and CD2 R: 5′-ggt cct tct cca gcc tag t-3′; Cxxc1 F: 5′-ggt tgt tgc acg ggg tcc ag-3′, Cxxc1 R: 5′-ccc cat tct cag act tgc tgt cg-3′; Ywhaz F: 5′-gtt act tgg ccg agg ttg ctg ct-3′, Ywhaz R: 5′-ggt gtg tcg gct gca tct cct t-3′. Cxxc1 and Ywhaz were used as reference genes. The ΔΔCq Analysis was performed with BioRad CFX Manager 3.1.

### Flow cytometry

Blood samples and splenocytes were treated with red blood cell lysis buffer and along with lymph node cells and thymocytes were washed with phosphate buffered saline (PBS). They were stained in FACS buffer (PBS, 10% FCS, 0.01% NaN_3_) with anti-CD2 FITC, anti-TCRβ PE, anti-CD8 PerCP, anti-CD19 APC and anti-CD4 PB (all Biolegend). Live/Dead discrimination was performed by propidium iodide or Live Dead Fixable Aqua Dead Cell Stain Kit (Life Technologies). Single cells were sorted (BD, MoFlow) onto AmpliGrid slides and processed immediately. For FACS analysis, cells were acquired at BD Canto II and Aria 5. Analysis was performed with FlowJo 9.4 and 10.

### Molecular Biology

Single cell PCR was performed as a nested PCR. The outer PCR was performed with Advantage2 (Clontech) directly on the AmpliGrid slide. For the inner PCR, Herculase II Fusion Enzyme with dNTP combos (Agilent) was used. PCRs were performed with the following primers: out fwd: 5′-atc acc ctg aac atc ccc aac-3′, out rev: 5′-act gga gtc ttc tt gtg ggc-3′ (product length: 382bp); in fwd: 5′-ctg gtc gca gag ttt aaa agg-3′, in rev: 5′-gct gct ccc caa ctt tct ac-3′ (product length 253bp). One AmpliGrid slide consists of 48 wells, of which every sixth sample was not loaded with a cell and served as negative control.

The Cas9-gRNA efficiency test of four different gRNAs targeting CD2 was performed *in vitro* on column-purified 839 bp CD2 PCR products (Wizard SV Gel and PCR Clean-UP System, Promega) which were amplified using Taq PCR Master Mix Kit (Qiagen) and following primers: CD2 cut F: 5′-cct gac aga aag aac tc-3′, CD2 cut R: 5′-ctc act gct cct agg ca-3′. Either PCR product or annealed oligos (PCR primer: T7 CD2.0 F: 5′-taa tac gac tca cta tag ggg act agg ctg gag aag gac c-3′, T7 CD2.0 R: 5′-gca gcg gct aaa aac gga-3′; oligos: T7 promoter F: 5′-taa tac gac tca cta tag gg-3′, CD2.1 R: 5′-aaa agc acc gac tcg gtg cca ctt ttt caa gtt gat aac gga cta gcc tta ttt taa ctt gct att tct agc tct aaa aca aga cac ccc aga tgg tct ccc cta tag tga gtc gta tta-3′, CD2.2 R: 5′-aaa agc acc gac tcg gtg cca ctt ttt caa gtt gat aac gga cta gcc tta ttt taa ctt gct att tct agc tct aaa acg aac atc ccc aac ttt caa acc cta tag tga gtc gta tta-3′, CD2.3 R: 5′- aaa agc acc gac tcg gtg cca ctt ttt caa gtt gat aac gga cta gcc tta ttt taa ctt gct att tct agc tct aaa act cgc acc tca tca ata tca tcc cta tag tga gtc gta tta-3′) were used to transcribe RNA with MEGAshortscript T7 Transcription Kit (ThermoFisher Scientific) and purified with MEGAclear Transcription Clean-Up Kit (ThermoFisher Scientific). gRNA and Cas9 Nuclease, S pyogenes (NEB) were preincubated for 10 min at room temperature. After adding the CD2 PCR product the mix was incubated for either 1 h or 1.5 h at 37 °C and finally analyzed on an agarose gel. The following fragment sizes are expected: gRNA(CD2.0): 499 bp and 340 bp, gRNA(CD2.1): 251 bp and 588 bp, gRNA(CD2.2): 269 bp and 570 bp, gRNA(CD2.3): 312 bp and 527 bp. The signal quantification was performed using ImageJ 1.50a.

## Additional Information

**How to cite this article**: Beil-Wagner, J. *et al.* T cell-specific inactivation of mouse CD2 by CRISPR/Cas9. *Sci. Rep.* **6**, 21377; doi: 10.1038/srep21377 (2016).

## Supplementary Material

Supplementary Information

## Figures and Tables

**Figure 2:**
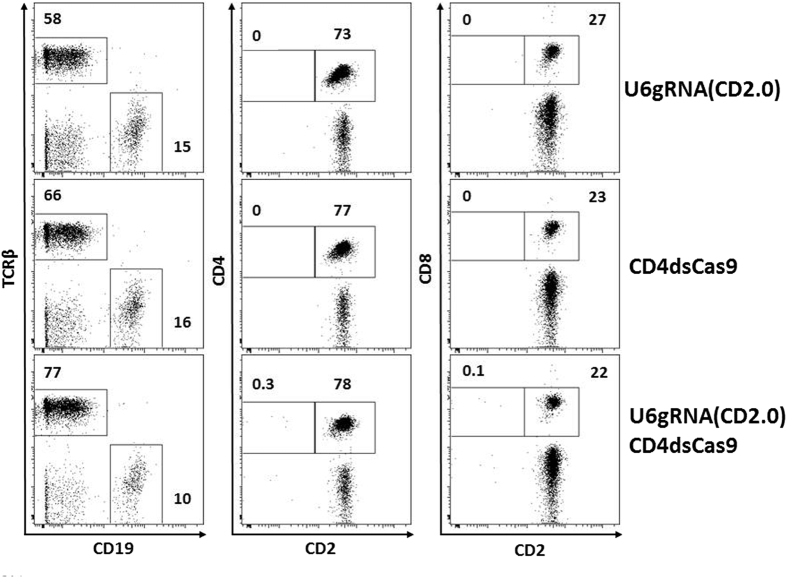
Abrogation of CD2 expression on a small population of peripheral blood T cells. Analysis of blood lymphocytes of the indicated transgenic mice by flow cytometry. Shown are live cells within a lymphocyte gate. CD4 and CD8 cells are additionally gated on TCRβ. The percentages of cells found within the marked gates of the dot plot analysis are shown.

**Figure 3:**
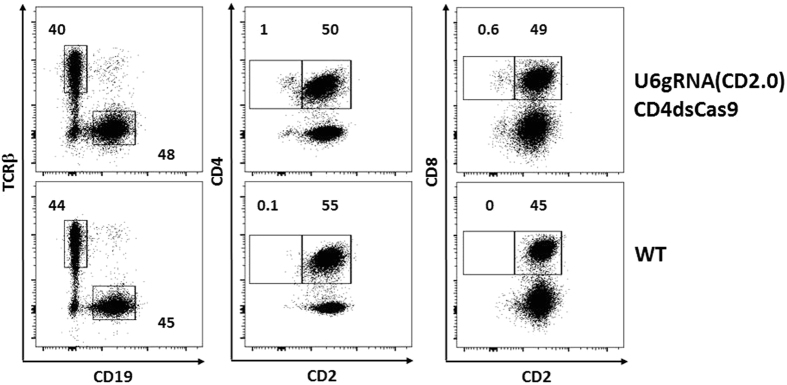
Abrogation of CD2 expression on a small population of lymph node/splenic T cells. Flow cytometric analysis of pooled lymphocytes from lymph nodes and spleens of two double transgenic and wildtype mice. Shown are live cells within a lymphocyte gate. The four plots on the right are additionally gated on TCRβ^+^ cells. The percentages of cells found within the marked gates of the dot plot analysis are indicated.

**Figure 4:**
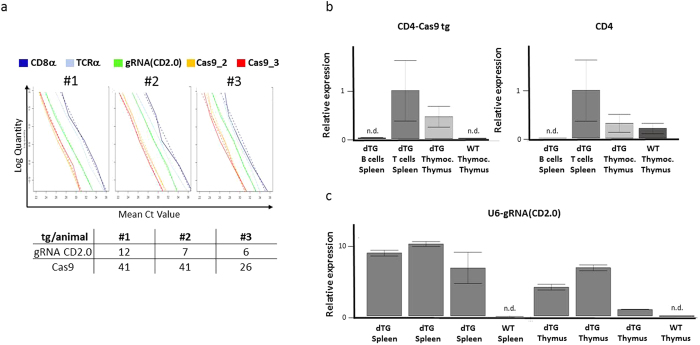
Analysis of transgene copy numbers and mRNA expression levels. (**a**) Serial dilutions of genomic DNA from three F1 offspring of founder #5 (Fig. 1c) were analyzed by real-time PCR specific for sequences within the indicated (trans)genes. A regression analysis was performed to calculate the copy number of the transgenes in each individual mouse, as indicated in the table. (**b**) Relative expression of CD4-Cas9 transgenic and endogenous CD4 mRNA. Transgenic splenic B cells, T cells and thymocytes as well as wildtype thymocytes were magnetically enriched and analyzed by RT-PCR. The CD4-Cas9 product is spanning the intron between CD4 Exon 1 and Cas9 ORF. Cxxc1 and Ywhaz served as housekeeping gene. Data was normalized to “dTG T cells Spleen”. (**c**) Relative expression of gRNA(CD2.0). Splenocytes and thymocytes of three different dTG offspring as well as of a wildtype control were analyzed by RT-PCR. Cxxc1 and Ywhaz served as housekeeping gene. Data was normalized to “dTG Thymus” of the third offspring.

**Figure 5:**
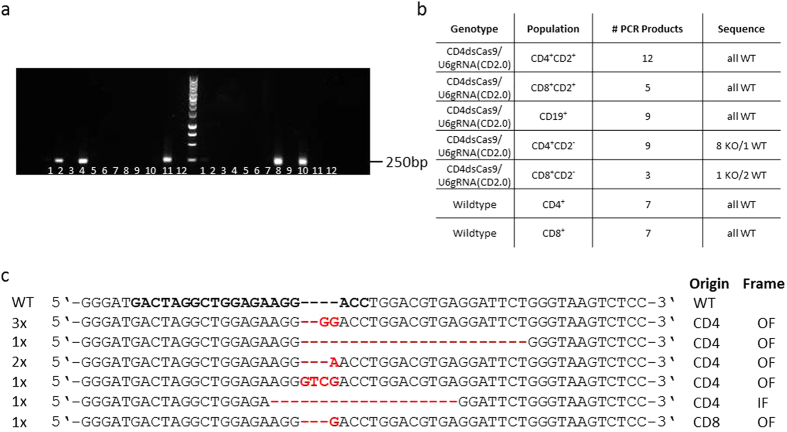
Single cell PCR analysis of the target region within the CD2 locus. Peripheral blood lymphocytes were surface stained for CD2, CD4, CD8, TCRβ and CD19 and the following populations within live cell and lymphocyte gates single cell-sorted by flow cytometry: TCRβ^+^ CD4^+^ CD2^+^, TCRβ^+^ CD8^+^ CD2^+^, TCRβ^+^ CD4^+^ CD2^−^, TCRβ^+^ CD8^+^ CD2^−^, CD19^+^. A 253 bp long region including the gRNA target was amplified by two rounds of nested PCR. Products were cloned in pGEM-T and pGEM-Teasy and sequenced. For wildtype controls the single cell PCR products were column-purified and sequenced directly. (**a**) Agarose gel showing PCR products of single cell amplicons of the target region within the CD2 gene locus from sorted peripheral blood single cells. Lanes 6 and 12 on both sides of the marker are H_2_0 negative controls. (**b**) Table showing the number of obtained mutations in amplicons of double-transgenic and wildtype cells. (**c**) Alignment of the obtained sequences from the CD2 gene amplicon. Indicated is the number (left side) and the cell type as well as the outcome of the mutation (OF: out of frame, IF: in frame) (right side) of the respective sequence. – indicates a deletion and red bases insertions.

**Figure 6:**
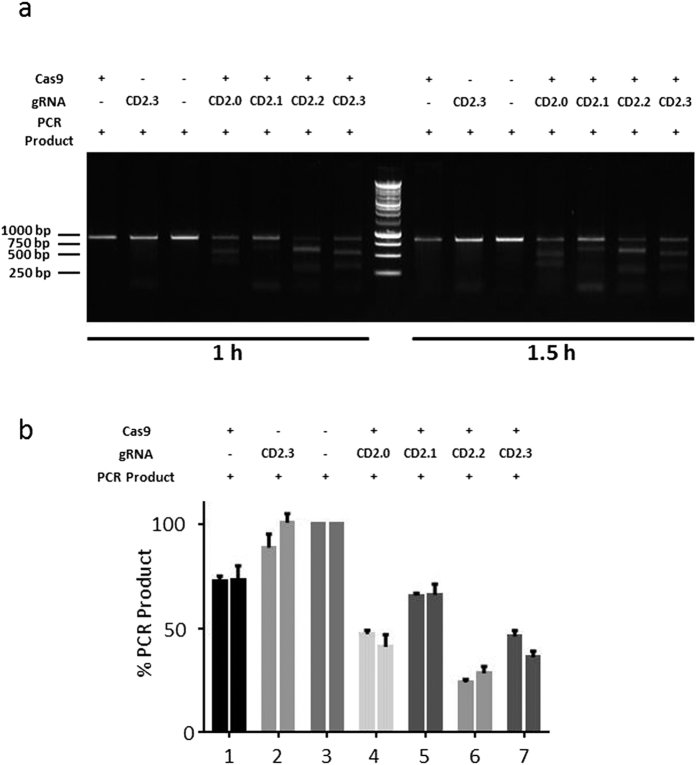
Functional analysis (**a**) *In vitro* test of gRNA efficiency. gRNA(CD2.0) as well as three controls (CD2.1,CD2.2, CD2.3) were incubated with a PCR product for the indicated period of time. Digests were separated on an agarose gel. (**b**) Analysis of the gRNA efficiency. The intensities of the bands resulting from gRNA/Cas9-digested PCR product of three different experiments were analyzed using ImageJ. Shown is the mean and standard error of the mean.
