# Supplementary material for: T cell-specific inactivation of mouse CD2 by CRISPR/Cas9
Source: Sci Rep. 2016 Feb 23;6:21377. doi: 10.1038/srep21377 (PMC4763270; doi:10.1038/srep21377)
Supplement: Supplementary Information [file srep21377-s1.pdf]

T cell-specific inactivation of mouse CD2 by CRISPR/Cas9

Jane Beil-Wagner[1,2], Georg Dössinger[1], Kilian Schober[1], Johannes vom Berg[2], Achim Tresch[3,4], Martina Grandl[1], Pushpalatha Palle[2], Florian Mair[5], Markus Gerhard[1], Burkhard Becher[5], Dirk H. Busch[1], Thorsten Buch[1,2]

[1]*Institute for Medical Microbiology, Immunology and Hygiene, Technische Universität München, Germany*

[2]*Institute of Laboratory Animal Science, University of Zurich, Schlieren, Switzerland*

[3]*Max-Planck-Institute for Plant Breeding Research, Cologne, Germany*

[4]*Department of Biology, Albertus-Magnus University, Cologne, Germany*

[5]*Institute of Experimental Immunology, University of Zurich, Zurich, Switzerland*

Correspondence to: Thorsten Buch
Institute of Laboratory Animal Science
University of Zurich
Wagistr. 12
8952 Schlieren
Switzerland
+41 44635 5473
thorsten.buch@uzh.ch

Figure Legends

Suppl. Fig. 1 Analysis of the non-mutating double-transgenic founder animal. Flow cytometric analysis of blood lymphocytes of the second founder carrying both transgenes (CD4dsCas9 and U6gRNA(CD2.0)). Shown are live cells within a lymphocyte gate.

Suppl. Fig. 2 Cell death analysis a) Analysis of blood lymphocytes of the indicated mouse strains by flow cytometry. Presented are cells lying in a lymphocyte gate. The percentage of cells found within the marked gates of the dot plot analysis are shown. Aqua live dead staining was used for cell death analysis. b) Analysis of pooled lymph nodes and spleen lymphocytes of two double transgenes as well as two wildtype littermates by flow cytometry. Presented are cells lying in a lymphocyte gate and are either CD19 or TCRβ positive. The percentage of cells found within the marked gates of the histogram analysis are shown. PI staining was used for cell death analysis.

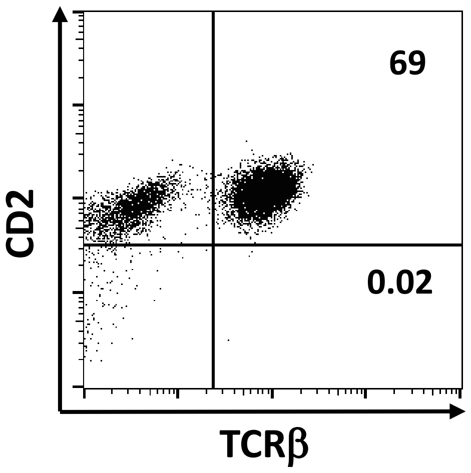
69
0.02
CD2
TCRβ

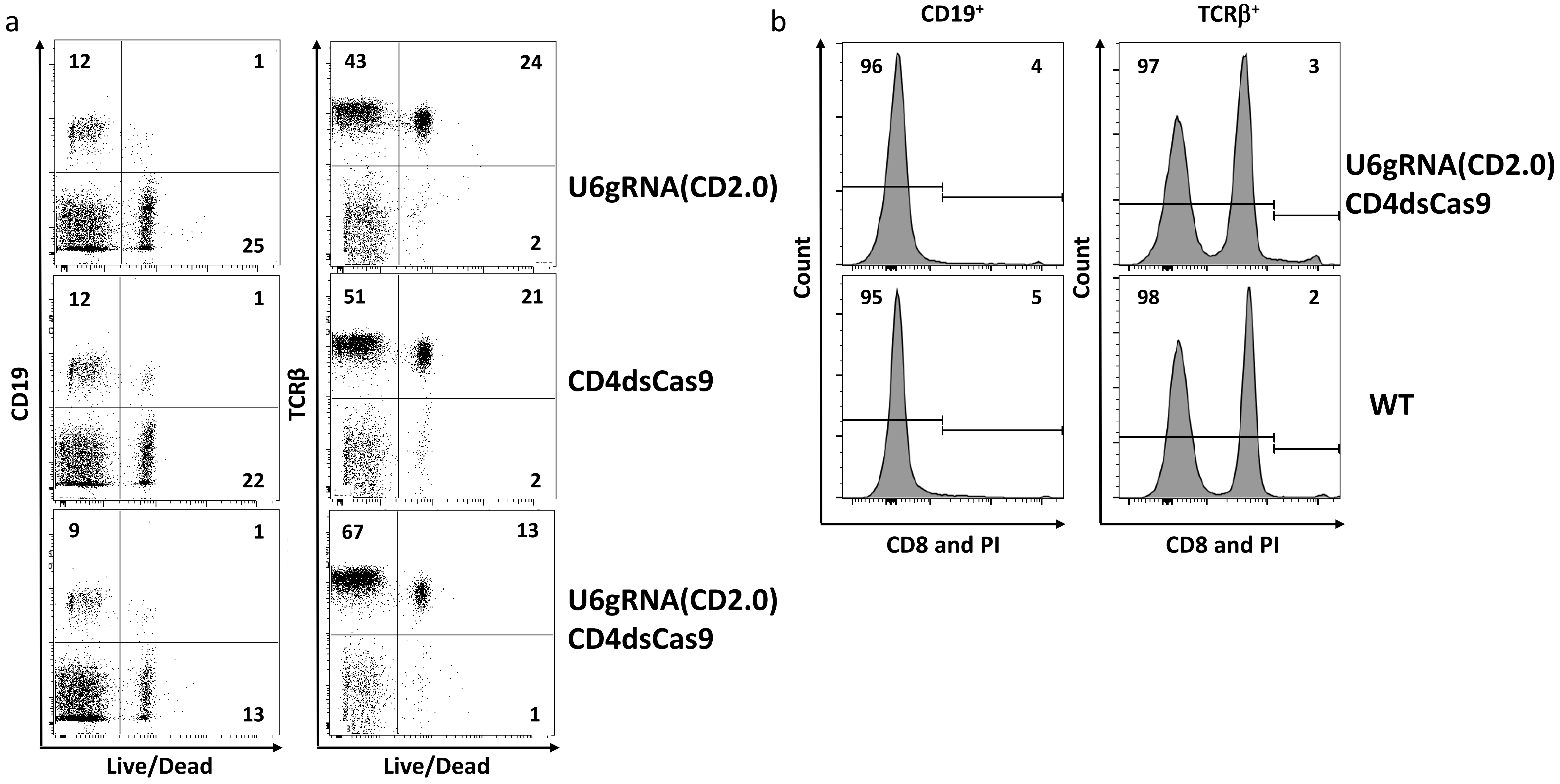
a
CD19
TCRβ
Live/Dead
Live/Dead
12
1
25
12
1
22
9
1
13
43
24
2
51
21
2
67
13
1
U6gRNA(CD2.0)
CD4dsCas9
U6gRNA(CD2.0)
CD4dsCas9
b
CD19+
TCRβ+
Count
Count
96
4
95
5
97
3
98
2
U6gRNA(CD2.0)
CD4dsCas9
WT
CD8 and PI
CD8 and PI
